# The genome sequence of the cabbage seedpod weevil, *Ceutorhynchus obstrictus* (Marsham, 1802) (Coleoptera: Curculionidae)

**DOI:** 10.12688/wellcomeopenres.26136.1

**Published:** 2026-03-18

**Authors:** Dmitry Telnov, Michael F. Geiser, Natural History Museum Genome Acquisition Lab, Darwin Tree of Life Barcoding Collective, Wellcome Sanger Institute Tree of Life Management, Samples and Laboratory team, Wellcome Sanger Institute Scientific Operations: Sequencing Operations, Wellcome Sanger Institute Tree of Life Core Informatics team, Tree of Life Core Informatics collective, Darwin Tree of Life Consortium

**Affiliations:** 1 Natural History Museum, London, England, UK; 2 Daugavpils University, Daugavpils, Latvia; 3 Institute of Biology University of Latvia, Rīga, Latvia

**Keywords:** Ceutorhynchus obstrictus; cabbage seedpod weevil; genome sequence; chromosomal; Coleoptera

## Abstract

We present a genome assembly from an individual male *Ceutorhynchus obstrictus* (cabbage seedpod weevil; Arthropoda; Insecta; Coleoptera; Curculionidae). The genome sequence has a total length of 728.81 megabases. Most of the assembly (93.83%) is scaffolded into 14 chromosomal pseudomolecules, including the X and Y sex chromosomes. The mitochondrial genome has also been assembled, with a length of 22.73 kilobases. Gene annotation of this assembly on Ensembl identified 15 692 protein-coding genes. This assembly was generated as part of the Darwin Tree of Life project, which produces reference genomes for eukaryotic species found in Britain and Ireland.

## Species taxonomy

Eukaryota; Opisthokonta; Metazoa; Eumetazoa; Bilateria; Protostomia; Ecdysozoa; Panarthropoda; Arthropoda; Mandibulata; Pancrustacea; Hexapoda; Insecta; Dicondylia; Pterygota; Neoptera; Endopterygota; Coleoptera; Polyphaga; Cucujiformia; Curculionoidea; Curculionidae; Ceutorhynchinae; *Ceutorhynchus*; *Ceutorhynchus obstrictus* (Marsham, 1802) (NCBI:txid307131).

## Background

The family Curculionidae Latreille, commonly known as weevils and bark beetles, is a hyperdiverse family of the order Coleoptera with over 51 000 species in more than 4 600 genera worldwide ( Anderson, 1995; Oberprieler *et al.*, 2007). This family comprises 14 subfamilies, and adults are morphologically heterogeneous. The family is of cosmopolitan distribution, most speciose in tropical regions. Adults and larvae of weevils (not including bark beetles) are phytophagous, occupying a wide variety of niches. Most of the species in the subfamily Ceutorhynchinae Gistel, 1848 (to which *Ceutorhynchus obstrictus* belongs) feed on crucifers (Brassicaceae) ( Morris, 2008). In the British fauna, approximately 500 species of weevils (including bark beetles) are currently known ( Duff, 2016, 2018). The genus *Ceutorhynchus* Germar, 1823 is represented by more than 130 species in the Palaearctic Region (e.g. Colonnelli, 2013) and 31 species in the British fauna ( Colonnelli, 2013; Duff, 2016).

*Ceutorhynchus obstrictus* (Marsham, 1802) is placed in the subfamily Ceutorhynchinae, tribe Ceutorhynchini ( Colonnelli, 2013). This species is also reported in the literature as *Ceutorhynchus assimilis* (auct. Non Paykull).

Adults are recognised by the longitudinal striae on the elytra, each bearing an embedded row of small, whitish, scale-like setae ( Duff, 2016). Males have a terminal spur on the meso- and metatibiae, which is absent in females; the male rostrum is shorter than that of females, and the prorostrum is 1.5–1.7× (males) and 1.9–2.0× (females) as long as the scape, with antennal insertion approximately at the midlength of the rostrum ( Morris, 2008). Genomic data may help to clarify species concepts and the origin of the British population of this abundant phytophagous beetle, which is sometimes considered a pest of brassica crops.

*Ceutorhynchus obstrictus* is a trans-Palaearctic species, widely distributed from the Iberian Peninsula and Ireland (where it is localised) throughout Türkiye, the Caucasus and Kazakhstan towards the Korean Peninsula and Mongolia; it has been introduced and established in North America ( Colonnelli, 2013; Morris, 2008). The species is not present in large areas of Palaearctic taiga. It is recorded from 35 European countries by Colonnelli (2013), including the UK but omitting Lithuania ( Tamutis *et al.*, 2011). The species is very abundant in Europe both in temperate and boreal zones but absent from subarctic regions. The European extent of occurrence and area of occupancy of this species both strongly exceed the thresholds for a threatened species ( GBIF, 2026) (also unpublished data by Telnov).

Both adults and larvae of *Ceutorhynchus obstrictus* are phytophagous, living in various open habitats such as arable fields, gardens and allotments and in all kinds of open and grassy places, including the sides of roads, tracks and paths, forest edges ( Morris, 2008). Adult weevils are reported to attack nearly all Brassicaceae genera ( Dieckmann, 1972). It is recorded as pests of cabbage, oilseed rape, turnip, radish and mustard crops ( Rheinheimer & Hassler, 2010 and references therein). Some plant species avoided by *Crabro obstrictus* are, for example, *Brassica nigra* and white mustard *Sinapis alba* ( Gratwick, 1992; Moyes & Raybould, 2001). Adults over-winter, emerge in early spring and start feeding on shoots, stems and flowers ( Dieckmann, 1972). Females lay eggs on young shoots from March to May, depending on the region. The larvae develop in fruit and feed on seeds ( Morris, 2008; Rheinheimer & Hassler, 2010). Pupation takes place in soil ( Rheinheimer & Hassler, 2010). Larval development takes a few months, and the new generation of adults emerges in late summer and early autumn, with a short feeding period before the winter diapause ( Rheinheimer & Hassler, 2010). The adult specimen used in the current study was sampled in July. Known parasites of *C. obstrictus* are several Braconidae, numerous Eulophidae ( Rheinheimer & Hassler, 2010). The species is documented as a serious pest of oilseed rape, while its effect on cabbage and other cultivated plants is less severe, since only seeds and not leaves are damaged ( Rheinheimer & Hassler, 2010).

In the United Kingdom, *C. obstrictus* is widespread through England, Wales and the Isle of Man, and more localised in Scotland and Northern Ireland ( Duff, 2016; GBIF, 2026). The northernmost records in the UK are from Kilmuir Easter and Logie Easter, Scotland ( GBIF, 2026). The species is not listed in the national Red Data Book ( Shirt, 1987). *Ceutorhynchus obstrictus* is one of the most abundant weevil species in Britain.

**Figure 1.**
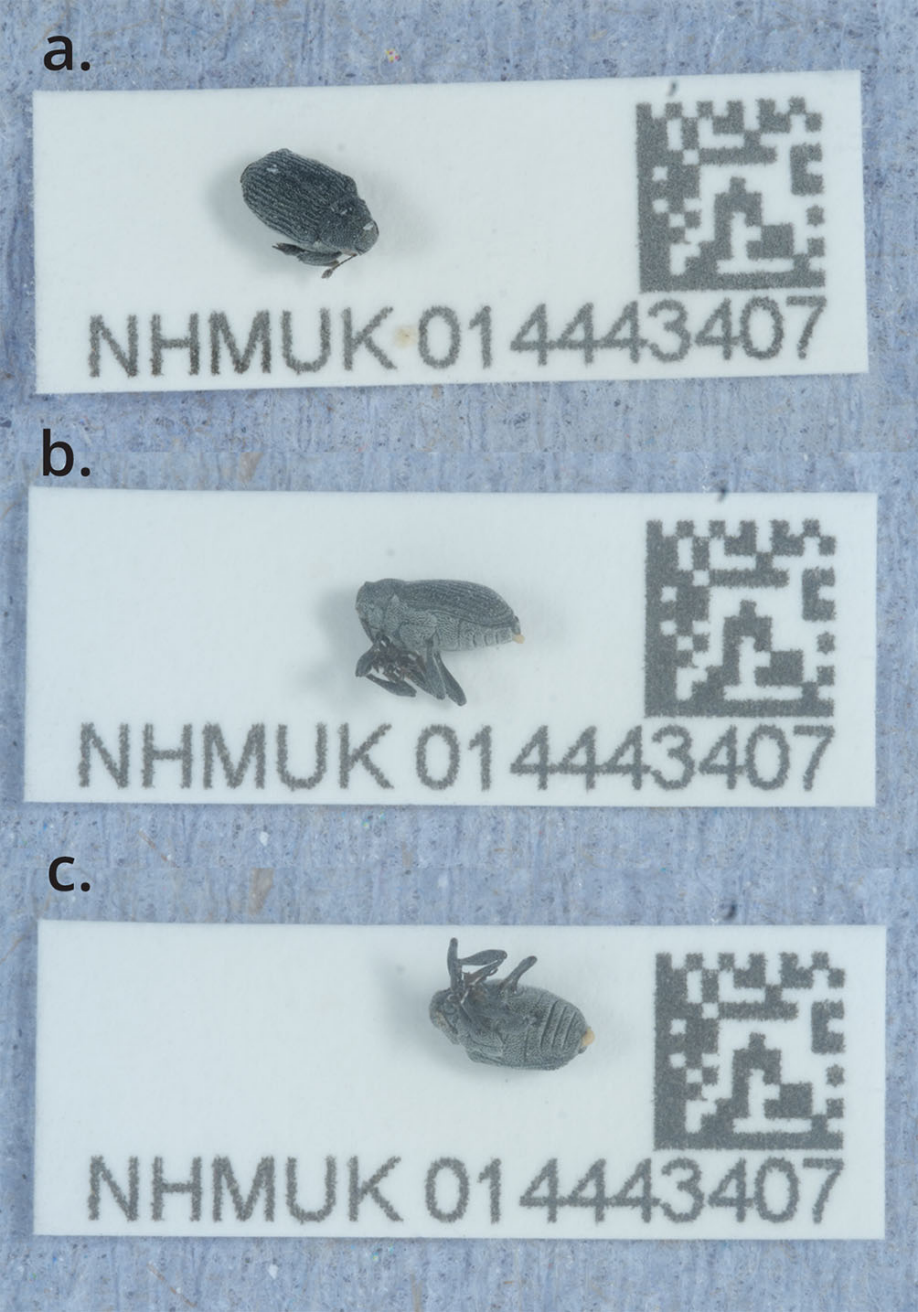
Photograph of the *Ceutorhynchus obstrictus* (icCeuObst1) specimen used for genome sequencing.

We present a chromosome-level genome sequence for *Ceutorhynchus obstrictus*, as part of the Darwin Tree of Life Project. The adult used for sequencing ( Figure 1) was collected in Winsford Gardens, Bromley, London, southern England.

## Methods

### Sample acquisition and DNA barcoding

The specimen used for genome sequencing was an adult male *Ceutorhynchus obstrictus* (specimen ID NHMUK014443407, ToLID icCeuObst1; Figure 1), collected from Penge, England, UK (latitude 51.41, longitude −0.06) on 2021-07-03. The specimen was collected and identified by Michael Geiser. A second specimen was used for Hi-C sequencing (specimen ID NHMUK013805911, ToLID icCeuObst2). It was collected from Hartslock Nature Reserve, England, UK (latitude 51.51, longitude −1.11) on 2021-07-29. The specimen was collected by Ian Sims and identified by Michael Geiser. Another third specimen, used for RNA sequencing (specimen ID NHMUK014440706, ToLID icCeuObst3), was collected from Wimbledon Common, England, UK (latitude 51.44, longitude −0.23) on 2022-03-06. The specimen was collected and identified by Michael Geiser. Details of the sampling and metadata procedures, which followed recommended standards, are described in Lawniczak *et al*. (2022).

The initial identification was verified by an additional DNA barcoding process according to the framework developed by Twyford *et al.* (2024). A small sample was dissected from the specimen and stored in ethanol, while the remaining parts were shipped on dry ice to the Wellcome Sanger Institute (WSI) (see the protocol). The tissue was lysed, the COI marker region was amplified by PCR, and amplicons were sequenced and compared to the BOLD database, confirming the species identification ( Crowley *et al.*, 2023). Following whole genome sequence generation, the relevant DNA barcode region was also used alongside the initial barcoding data for sample tracking at the WSI ( Twyford *et al.*, 2024). The standard operating procedures for Darwin Tree of Life barcoding are available on protocols.io.

### Nucleic acid extraction

Protocols for high molecular weight (HMW) DNA extraction developed at the Wellcome Sanger Institute (WSI) Tree of Life Core Laboratory are available on protocols.io ( Howard *et al.*, 2025). The icCeuObst1 sample was weighed and triaged to determine the appropriate extraction protocol. Tissue from the whole organism was homogenised by powermashing using a PowerMasher II tissue disruptor.

HMW DNA was extracted in the WSI Scientific Operations core using the MagAttract protocol. DNA was sheared into an average fragment size of 12–20 kb following the Megaruptor ^®^3 for LI PacBio protocol. Sheared DNA was purified by automated SPRI (solid-phase reversible immobilisation). The concentration of the sheared and purified DNA was assessed using a Nanodrop spectrophotometer and Qubit Fluorometer using the Qubit dsDNA High Sensitivity Assay kit. Fragment size distribution was evaluated by running the sample on the FemtoPulse system. For this sample, the final post-shearing DNA had a Qubit concentration of 9.46 ng/μL and a yield of 450.30 ng, with a fragment size of 16.5 kb.

RNA was extracted from whole organism tissue of icCeuObst3 in the Tree of Life Laboratory at the WSI using the RNA Extraction: Automated MagMax™ *mir*Vana protocol. The RNA concentration was assessed using a Nanodrop spectrophotometer and a Qubit Fluorometer using the Qubit RNA Broad-Range Assay kit. Analysis of the integrity of the RNA was done using the Agilent RNA 6000 Pico Kit and Eukaryotic Total RNA assay.

### PacBio HiFi library preparation and sequencing

Library preparation and sequencing were performed at the WSI Scientific Operations core. Libraries were prepared using the SMRTbell Prep Kit 3.0 (Pacific Biosciences, California, USA), following the manufacturer’s instructions. The kit includes reagents for end repair/A-tailing, adapter ligation, post-ligation SMRTbell bead clean-up, and nuclease treatment. Size selection and clean-up were performed using diluted AMPure PB beads (Pacific Biosciences). DNA concentration was quantified using a Qubit Fluorometer v4.0 (ThermoFisher Scientific) and the Qubit 1X dsDNA HS assay kit. Final library fragment size was assessed with the Agilent Femto Pulse Automated Pulsed Field CE Instrument (Agilent Technologies) using the gDNA 55 kb BAC analysis kit.

The sample was sequenced using the Sequel IIe system (Pacific Biosciences, California, USA). The concentration of the library loaded onto the Sequel IIe was in the range 40–135 pM. The SMRT link software, a PacBio web-based end-to-end workflow manager, was used to set-up and monitor the run, and to perform primary and secondary analysis of the data upon completion.

### Hi-C

***Sample preparation and crosslinking***

The Hi-C sample was prepared from 20–50 mg of frozen tissue from the icCeuObst2 sample using the Arima-HiC v2 kit (Arima Genomics). Following the manufacturer’s instructions, tissue was fixed and DNA crosslinked using TC buffer to a final formaldehyde concentration of 2%. The tissue was homogenised using the Diagnocine Power Masher-II. Crosslinked DNA was digested with a restriction enzyme master mix, biotinylated, and ligated. Clean-up was performed with SPRISelect beads before library preparation. DNA concentration was measured with the Qubit Fluorometer (Thermo Fisher Scientific) and Qubit HS Assay Kit. The biotinylation percentage was estimated using the Arima-HiC v2 QC beads.

***Hi-C library preparation and sequencing***

Biotinylated DNA constructs were fragmented using a Covaris E220 sonicator and size selected to 400–600 bp using SPRISelect beads. DNA was enriched with Arima-HiC v2 kit Enrichment beads. End repair, A-tailing, and adapter ligation were carried out with the NEBNext Ultra II DNA Library Prep Kit (New England Biolabs), following a modified protocol where library preparation occurs while DNA remains bound to the Enrichment beads. Library amplification was performed using KAPA HiFi HotStart mix and a custom Unique Dual Index (UDI) barcode set (Integrated DNA Technologies). Depending on sample concentration and biotinylation percentage determined at the crosslinking stage, libraries were amplified with 10–16 PCR cycles. Post-PCR clean-up was performed with SPRISelect beads. Libraries were quantified using the AccuClear Ultra High Sensitivity dsDNA Standards Assay Kit (Biotium) and a FLUOstar Omega plate reader (BMG Labtech).

Prior to sequencing, libraries were normalised to 10 ng/μL. Normalised libraries were quantified again to create equimolar and/or weighted 2.8 nM pools. Pool concentrations were checked using the Agilent 4200 TapeStation (Agilent) with High Sensitivity D500 reagents before sequencing. Sequencing was performed using paired-end 150 bp reads on the Illumina NovaSeq 6000.

### RNA library preparation and sequencing

Libraries were prepared using the NEBNext ^®^ Ultra™ II Directional RNA Library Prep Kit for Illumina (New England Biolabs), following the manufacturer’s instructions. Poly(A) mRNA in the total RNA solution was isolated using oligo (dT) beads, converted to cDNA, and uniquely indexed; 14 PCR cycles were performed. Libraries were size-selected to produce fragments between 100–300 bp. Libraries were quantified, normalised, pooled to a final concentration of 2.8 nM, and diluted to 150 pM for loading. Sequencing was carried out on the Illumina NovaSeq X to generate 150-bp paired-end reads.

### Genome assembly

Prior to assembly of the PacBio HiFi reads, a database of *k*-mer counts ( *k* = 31) was generated from the filtered reads using FastK. GenomeScope2 ( Ranallo-Benavidez *et al.*, 2020) was used to analyse the *k*-mer frequency distributions, providing estimates of genome size, heterozygosity, and repeat content.

The HiFi reads were assembled using Hifiasm ( Cheng *et al.*, 2021) with the --primary option. Haplotypic duplications were identified and removed using purge_dups ( Guan *et al.*, 2020). The Hi-C reads ( Rao *et al.*, 2014) were mapped to the primary contigs using bwa-mem2 ( Vasimuddin *et al.*, 2019), and the contigs were scaffolded in YaHS ( Zhou *et al.*, 2023) with the --break option for handling potential misassemblies. The scaffolded assemblies were evaluated using Gfastats ( Formenti *et al.*, 2022), BUSCO ( Manni *et al.*, 2021) and MERQURY.FK ( Rhie *et al.*, 2020).

The mitochondrial genome was assembled using MitoHiFi ( Uliano-Silva *et al.*, 2023), which runs MitoFinder ( Allio *et al.*, 2020) and uses these annotations to select the final mitochondrial contig and to ensure the general quality of the sequence.

### Assembly curation

The assembly was decontaminated using the Assembly Screen for Cobionts and Contaminants ( ASCC) pipeline. TreeVal was used to generate the flat files and maps for use in curation. Manual curation was conducted primarily in PretextView and HiGlass ( Kerpedjiev *et al.*, 2018). Scaffolds were visually inspected and corrected as described by Howe *et al*. (2021). Manual corrections included three breaks and 20 joins. This reduced the scaffold count by 8.5% and increased the scaffold N50 by 64.1%. The curation process is documented at https://gitlab.com/wtsi-grit/rapid-curation . PretextSnapshot was used to generate a Hi-C contact map of the final assembly.

### Assembly quality assessment

The Merqury.FK tool ( Rhie *et al.*, 2020) was run in a Singularity container ( Kurtzer *et al.*, 2017) to evaluate *k*-mer completeness and assembly quality for the primary and alternate haplotypes using the *k*-mer database ( *k* = 31) computed prior to genome assembly. The analysis outputs included assembly QV scores and completeness statistics.

The genome was analysed using the BlobToolKit pipeline, a Nextflow implementation of the earlier Snakemake version ( Challis *et al.*, 2020). The pipeline aligns PacBio reads using minimap2 ( Li, 2018) and SAMtools ( Danecek *et al.*, 2021) to generate coverage tracks. It runs BUSCO ( Manni *et al.*, 2021) using lineages identified from the NCBI Taxonomy ( Schoch *et al.*, 2020). For the three domain-level lineages, BUSCO genes are aligned to the UniProt Reference Proteomes database ( Bateman *et al.*, 2023) using DIAMOND blastp ( Buchfink *et al.*, 2021). The genome is divided into chunks based on the density of BUSCO genes from the closest taxonomic lineage, and each chunk is aligned to the UniProt Reference Proteomes database with DIAMOND blastx. Sequences without hits are chunked using seqtk and aligned to the NT database with blastn ( Altschul *et al.*, 1990). The BlobToolKit suite consolidates all outputs into a blobdir for visualisation. The BlobToolKit pipeline was developed using nf-core tooling ( Ewels *et al.*, 2020) and MultiQC ( Ewels *et al.*, 2016), with containerisation through Docker ( Merkel, 2014) and Singularity ( Kurtzer *et al.*, 2017).

## Genome sequence report

### Sequence data

**Figure 2.**
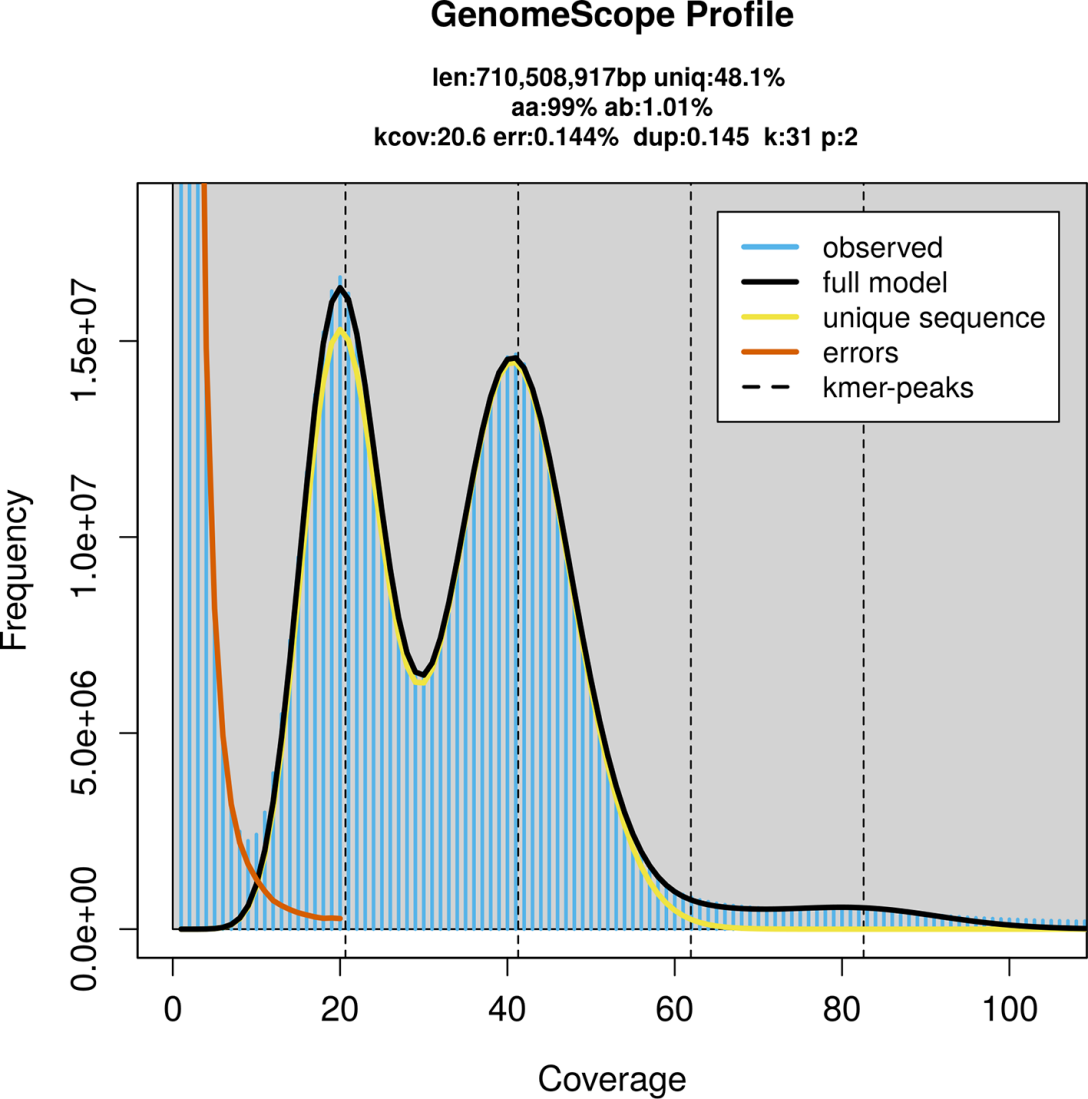
Frequency distribution of *k*-mers generated using GenomeScope2. The plot shows observed and modelled *k*-mer spectra, providing estimates of genome size, heterozygosity, and repeat content based on unassembled sequencing reads.

PacBio sequencing of the *Ceutorhynchus obstrictus* specimen generated 30.74 Gb (gigabases) from 2.45 million reads, which were used to assemble the genome. GenomeScope2.0 analysis estimated the haploid genome size at 710.51 Mb, with a heterozygosity of 1.01% and repeat content of 52.11% ( Figure 2). These estimates guided expectations for the assembly. Based on the estimated genome size, the sequencing data provided approximately 41× coverage. Hi-C sequencing produced 192.95 Gb from 1 277.82 million reads, which were used to scaffold the assembly. RNA sequencing data were also generated and are available in public sequence repositories. Table 1 summarises the specimen and sequencing details.

**Table 1.** Specimen and sequencing data for BioProject PRJEB59808.

Platform	PacBio HiFi	Hi-C	RNA-seq
**ToLID**	icCeuObst1	icCeuObst2	icCeuObst3
**Specimen ID**	NHMUK014443407	NHMUK013805911	NHMUK014440706
**BioSample (source individual)**	SAMEA111458184	SAMEA111458440	SAMEA114805995
**BioSample (tissue)**	SAMEA111458273	SAMEA111458496	SAMEA114806195
**Tissue**	whole organism	whole organism	whole organism
**Instrument**	Sequel IIe	Illumina NovaSeq 6000	Illumina NovaSeq X
**Run accessions**	ERR10879943	ERR10890756	ERR13493904
**Read count total**	2.45 million	1 277.82 million	118.68 million
**Base count total**	30.74 Gb	192.95 Gb	17.92 Gb

### Assembly statistics

The primary haplotype was assembled, and contigs corresponding to an alternate haplotype were also deposited in INSDC databases. The final assembly has a total length of 728.81 Mb in 193 scaffolds, with 243 gaps, and a scaffold N50 of 56.42 Mb ( Table 2).

**Table 2.** Genome assembly statistics.

**Assembly name**	icCeuObst1.1
**Assembly accession**	GCA_965178145.1
**Alternate haplotype accession**	GCA_965178005.1
**Assembly level**	chromosome
**Span (Mb)**	728.81
**Number of chromosomes**	14
**Number of contigs**	436
**Contig N50**	4.75 Mb
**Number of scaffolds**	193
**Scaffold N50**	56.42 Mb
**Sex chromosomes**	X and Y
**Organelles**	Mitochondrion: 22.73 kb

**Figure 3.**
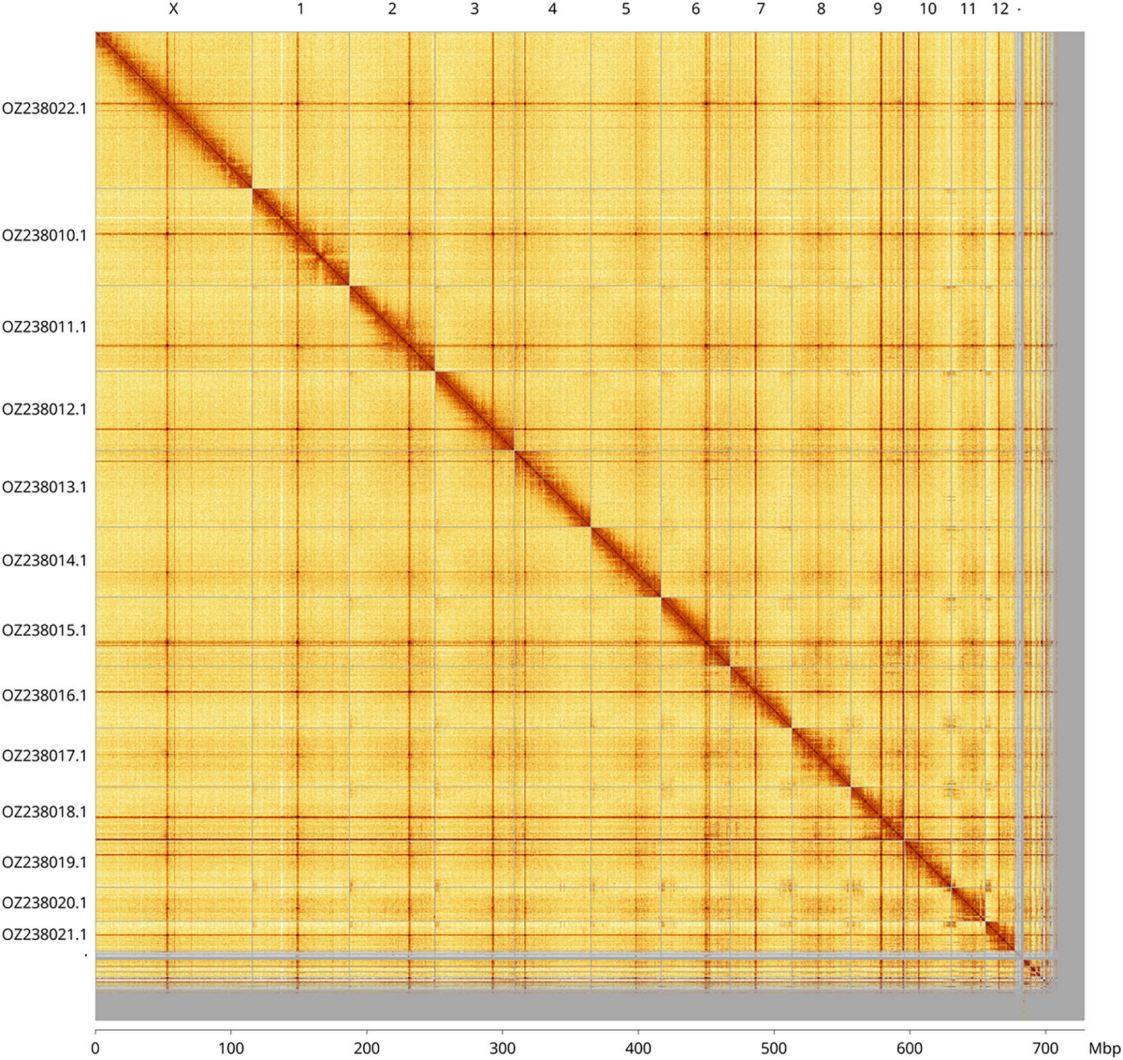
Hi-C contact map of the *Ceutorhynchus obstrictus* genome assembly. Assembled chromosomes are shown in order of size and labelled along the axes, with a megabase scale shown below. The plot was generated using PretextSnapshot.

Most of the assembly sequence (93.83%) was assigned to 14 chromosomal-level scaffolds, representing 12 autosomes and the X and Y sex chromosomes. These chromosome-level scaffolds, confirmed by Hi-C data, are named according to size ( Figure 3; Table 3). Chromosomes X and Y were identified by pacbio read coverage and Hi-C signal.

**Table 3.** Chromosomal pseudomolecules in the primary genome assembly of *Ceutorhynchus obstrictus* icCeuObst1.

INSDC accession	Molecule	Length (Mb)	GC%
OZ238010.1	1	71.62	35.50
OZ238011.1	2	63.25	35
OZ238012.1	3	58.25	35.50
OZ238013.1	4	56.42	35.50
OZ238014.1	5	51.84	35
OZ238015.1	6	50.72	35.50
OZ238016.1	7	45.37	35
OZ238017.1	8	43.56	35
OZ238018.1	9	39.36	35
OZ238019.1	10	34.67	35
OZ238020.1	11	24.97	35
OZ238021.1	12	21.90	34.50
OZ238022.1	X	115.60	35.50
OZ238023.1	Y	6.29	35.50

The mitochondrial genome was also assembled (length 22.73 kb, OZ238024.1). This sequence is included as a contig in the multifasta file of the genome submission and as a standalone record.

**Figure 4.**
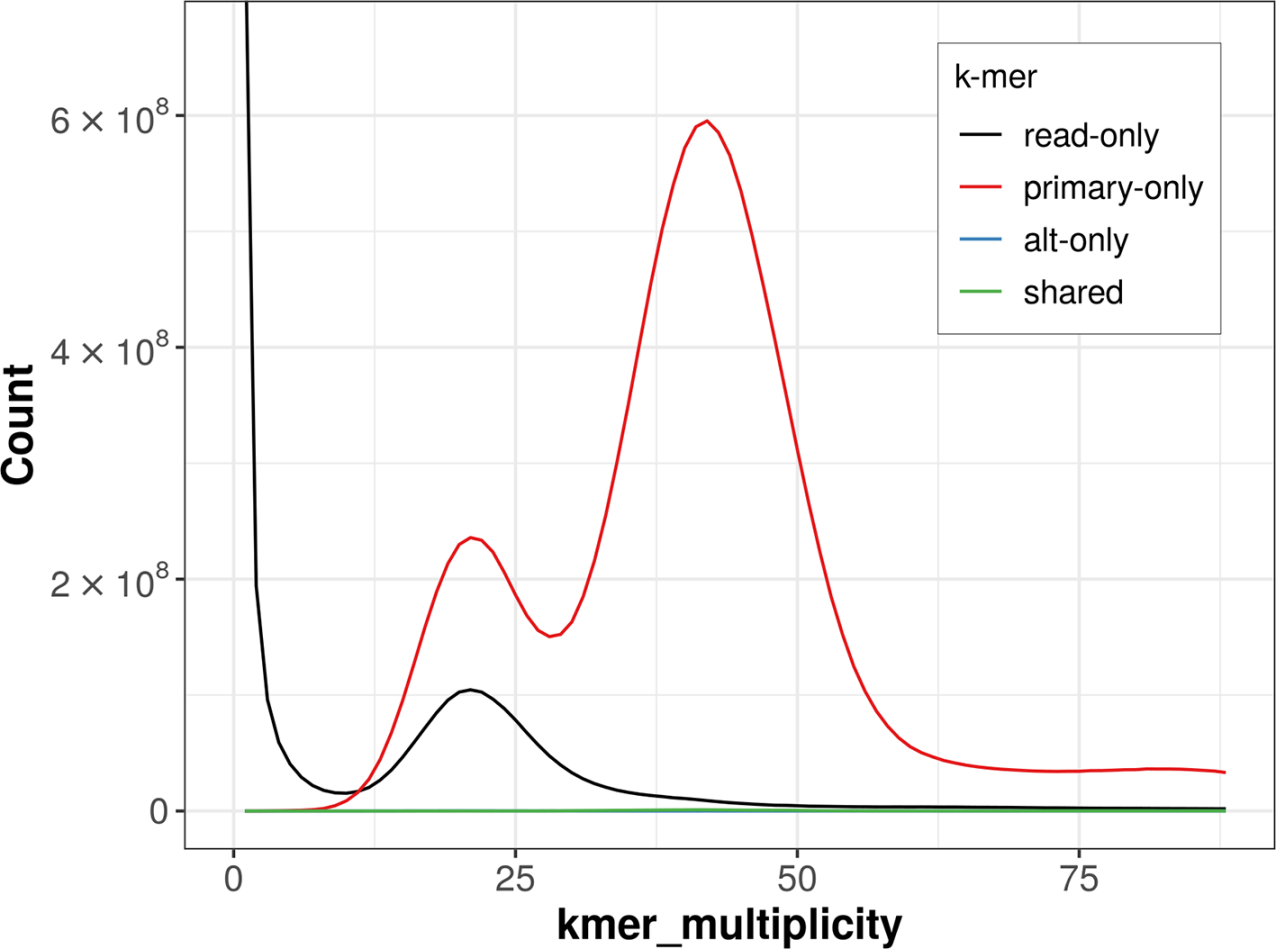
Evaluation of *k*-mer completeness using MerquryFK. This plot illustrates the recovery of *k*-mers from the original read data in the final assemblies. The horizontal axis represents *k*-mer multiplicity, and the vertical axis shows the number of *k*-mers. The black curve represents *k*-mers that appear in the reads but are not assembled. The green curve corresponds to *k*-mers shared by both haplotypes, and the red and blue curves show *k*-mers found only in one of the haplotypes.

The combined primary and alternate assemblies achieve an estimated QV of 65.8. The *k*-mer completeness is 84.65% for the primary assembly, 0.20% for the alternate haplotype, and 84.67% for the combined assemblies ( Figure 4).

**Figure 5.**
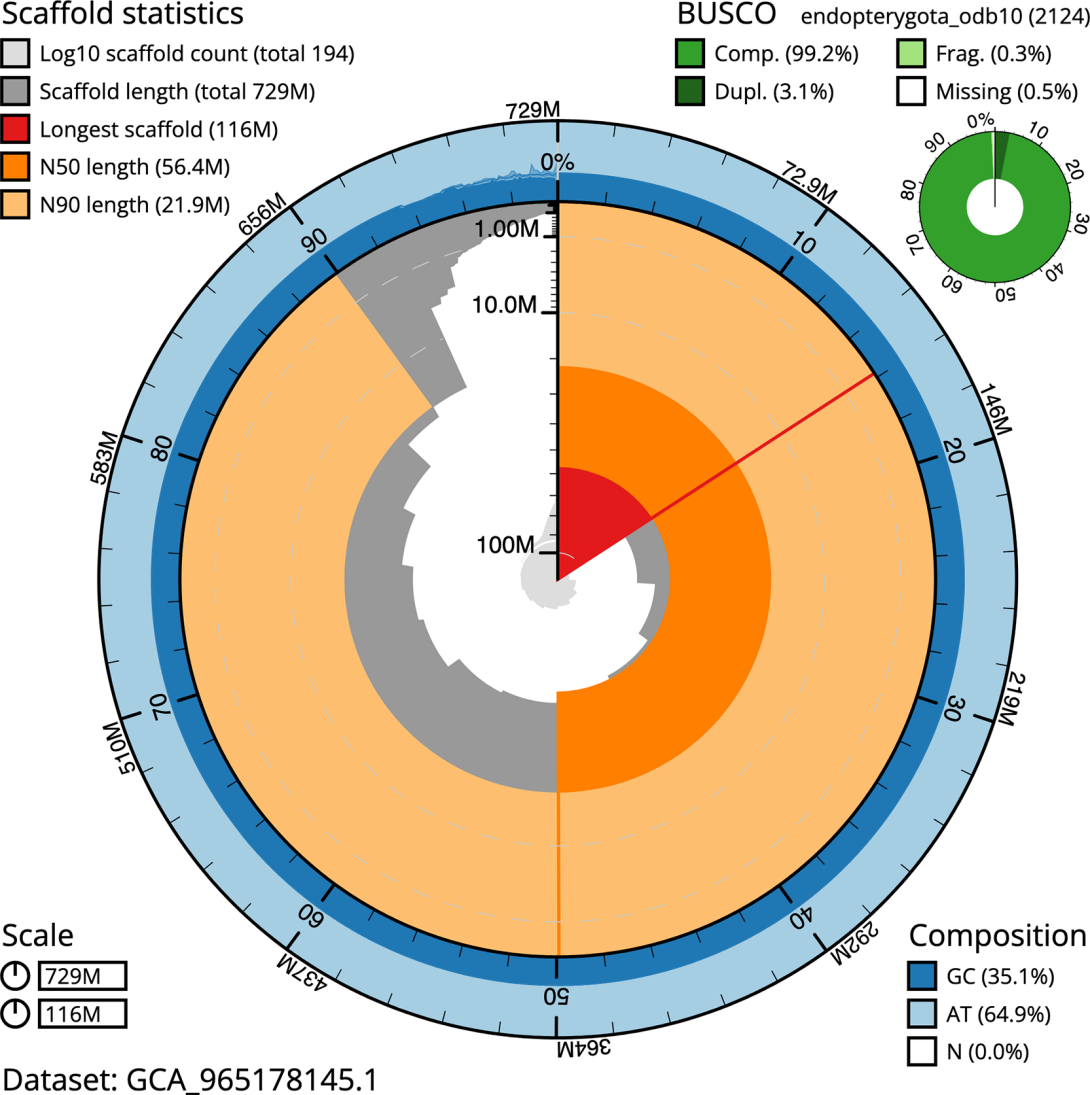
Assembly metrics for icCeuObst1.1. The BlobToolKit snail plot provides an overview of assembly metrics and BUSCO gene completeness. The circumference represents the length of the whole genome sequence, and the main plot is divided into 1 000 bins around the circumference. The outermost blue tracks display the distribution of GC, AT, and N percentages across the bins. Scaffolds are arranged clockwise from longest to shortest and are depicted in dark grey. The longest scaffold is indicated by the red arc, and the deeper orange and pale orange arcs represent the N50 and N90 lengths. A light grey spiral at the centre shows the cumulative scaffold count on a logarithmic scale. A summary of complete, fragmented, duplicated, and missing BUSCO genes in the endopterygota_odb10 set is presented at the top right. An interactive version of this figure can be accessed on the BlobToolKit viewer.

**Figure 6.**
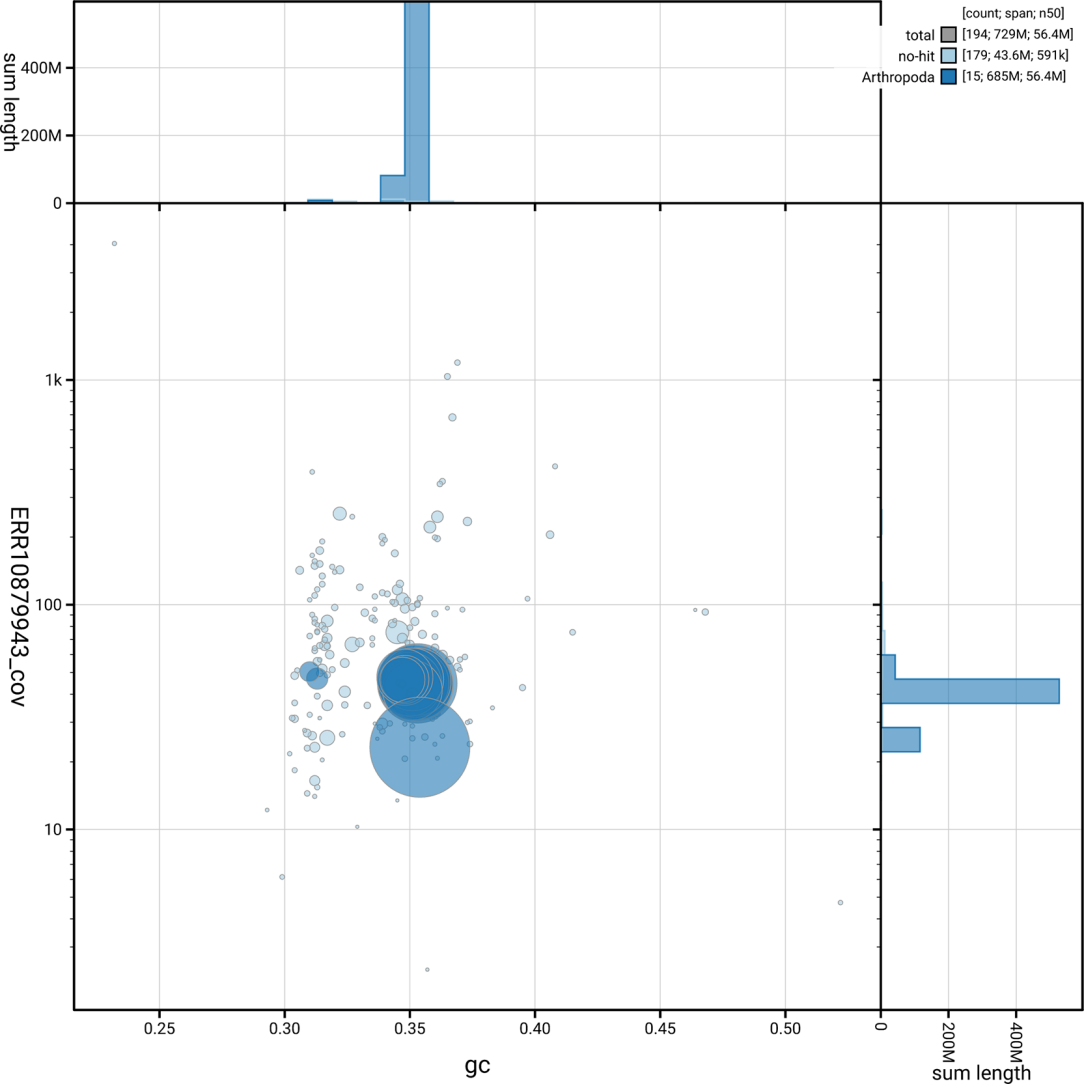
BlobToolKit GC-coverage plot for icCeuObst1.1. Blob plot showing sequence coverage (vertical axis) and GC content (horizontal axis). The circles represent scaffolds, with the size proportional to scaffold length and the colour representing phylum membership. The histograms along the axes display the total length of sequences distributed across different levels of coverage and GC content. An interactive version of this figure is available on the BlobToolKit viewer.

BUSCO v.5.7.1 analysis using the endopterygota_odb10 reference set ( *n* = 2 124) identified 99.2% of the expected gene set (single = 96.1%, duplicated = 3.1%). The snail plot in Figure 5 summarises the scaffold length distribution and other assembly statistics for the primary assembly. The blob plot in Figure 6 shows the distribution of scaffolds by GC proportion and coverage.

Table 4 lists the assembly metric benchmarks adapted from Rhie *et al.* (2021) and the Earth BioGenome Project Report on Assembly Standards September 2024. The EBP metric, calculated for the primary assembly, is **6.C.Q65**, meeting the recommended reference standard.

**Table 4.** Earth Biogenome Project summary metrics for the *Ceutorhynchus obstrictus* assembly.

Measure	Value	Benchmark
EBP summary (primary)	6.C.Q65	6.C.Q40
Contig N50 length	4.75 Mb	≥ 1 Mb
Scaffold N50 length	56.42 Mb	= chromosome N50
Consensus quality (QV)	Primary: 65.8; alternate: inf; combined: 65.8	≥ 40
*k*-mer completeness	Primary: 84.65%; alternate: 0.20%; combined: 84.67%	≥ 95%
BUSCO	C:99.2% [S:96.1%; D:3.1%]; F:0.3%; M:0.5%; n:2 124	S > 90%; D < 5%
Percentage of assembly assigned to chromosomes	93.83%	≥ 90%

**Table 5.** Software versions and sources.

Software	Version	Source
BEDTools	2.30.0	https://github.com/arq5x/bedtools2
BLAST	2.14.0	ftp://ftp.ncbi.nlm.nih.gov/blast/executables/blast+/
BlobToolKit	4.4.5	https://github.com/blobtoolkit/blobtoolkit
BUSCO	5.7.1	https://gitlab.com/ezlab/busco
bwa-mem2	2.2.1	https://github.com/bwa-mem2/bwa-mem2
Cooler	0.8.11	https://github.com/open2c/cooler
DIAMOND	2.1.8	https://github.com/bbuchfink/diamond
fasta_windows	0.2.4	https://github.com/tolkit/fasta_windows
FastK	1.1	https://github.com/thegenemyers/FASTK
GenomeScope2.0	2.0.1	https://github.com/tbenavi1/genomescope2.0
Gfastats	1.3.6	https://github.com/vgl-hub/gfastats
GoaT CLI	0.2.5	https://github.com/genomehubs/goat-cli
Hifiasm	0.16.1-r375	https://github.com/chhylp123/hifiasm
HiGlass	1.13.4	https://github.com/higlass/higlass
MerquryFK	1.1.2	https://github.com/thegenemyers/MERQURY.FK
Minimap2	2.28-r1209	https://github.com/lh3/minimap2
MitoHiFi	2	https://github.com/marcelauliano/MitoHiFi
MultiQC	1.14; 1.17 and 1.18	https://github.com/MultiQC/MultiQC
Nextflow	24.10.4	https://github.com/nextflow-io/nextflow
PretextSnapshot	0.0.5	https://github.com/sanger-tol/PretextSnapshot
PretextView	0.2.5	https://github.com/sanger-tol/PretextView
purge_dups	1.2.3	https://github.com/dfguan/purge_dups
samtools	1.21	https://github.com/samtools/samtools
sanger-tol/ascc	0.1.0	https://github.com/sanger-tol/ascc
sanger-tol/blobtoolkit	v0.7.1	https://github.com/sanger-tol/blobtoolkit
sanger-tol/curationpretext	1.4.2	https://github.com/sanger-tol/curationpretext
Seqtk	1.3	https://github.com/lh3/seqtk
Singularity	3.9.0	https://github.com/sylabs/singularity
TreeVal	1.4.0	https://github.com/sanger-tol/treeval
YaHS	1.2a	https://github.com/c-zhou/yahs

### Genome annotation report

The *Ceutorhynchus obstrictus* genome assembly (GCA_965178145.1) was annotated by Ensembl at the European Bioinformatics Institute (EBI), using the Ensembl non-vertebrate genome annotation system. This annotation includes 30 834 transcribed mRNAs from 15 692 protein-coding and 4 206 non-coding genes. The average transcript length is 19 009.58 bp, with an average of 1.55 coding transcripts per gene and 5.81 exons per transcript. For further information about the annotation, please refer to the annotation page on Ensembl.

## Author information

Contributors are listed at the following links:

• Members of the University of Oxford and Wytham Woods Genome Acquisition Lab
• Members of the Natural History Museum Genome Acquisition Lab
• Members of the Marine Biological Association Genome Acquisition Lab
• Members of the Darwin Tree of Life Barcoding collective
• Members of the Wellcome Sanger Institute Tree of Life Management, Samples and Laboratory team
• Members of Wellcome Sanger Institute Scientific Operations – Sequencing Operations
• Members of the Wellcome Sanger Institute Tree of Life Core Informatics team
• Members of the Tree of Life Core Informatics collective
• Members of the Darwin Tree of Life Consortium

## Wellcome Sanger Institute – Legal and governance

The materials that have contributed to this genome note have been supplied by a Darwin Tree of Life Partner. The submission of materials by a Darwin Tree of Life Partner is subject to the **‘Darwin Tree of Life Project Sampling Code of Practice’**, which can be found in full on the Darwin Tree of Life website. By agreeing with and signing up to the Sampling Code of Practice, the Darwin Tree of Life Partner agrees they will meet the legal and ethical requirements and standards set out within this document in respect of all samples acquired for, and supplied to, the Darwin Tree of Life Project. Further, the Wellcome Sanger Institute employs a process whereby due diligence is carried out proportionate to the nature of the materials themselves, and the circumstances under which they have been/are to be collected and provided for use. The purpose of this is to address and mitigate any potential legal and/or ethical implications of receipt and use of the materials as part of the research project, and to ensure that in doing so we align with best practice wherever possible. The overarching areas of consideration are:

• Ethical review of provenance and sourcing of the material
• Legality of collection, transfer and use (national and international)

Each transfer of samples is further undertaken according to a Research Collaboration Agreement or Material Transfer Agreement entered into by the Darwin Tree of Life Partner, Genome Research Limited (operating as the Wellcome Sanger Institute), and in some circumstances, other Darwin Tree of Life collaborators.

## Data Availability

European Nucleotide Archive: Ceutorhynchus obstrictus. Accession number PRJEB59808. The genome sequence is released openly for reuse. The *Ceutorhynchus obstrictus* genome sequencing initiative is part of the Darwin Tree of Life Project (PRJEB40665) and the Sanger Institute Tree of Life Programme (PRJEB43745). All raw sequence data and the assembly have been deposited in INSDC databases. Raw data and assembly accession identifiers are reported in Tables 1 and 2. Production code used in genome assembly at the WSI Tree of Life is available at https://github.com/sanger-tol . Table 5 lists software versions used in this study.
