# Supplementary material for: The genome sequence of the cabbage seedpod weevil, Ceutorhynchus obstrictus (Marsham, 1802) (Coleoptera: Curculionidae)
Source: Wellcome Open Res. 2026 Mar 18;11:172. [Version 1] doi: 10.12688/wellcomeopenres.26136.1 (PMC13153772; doi:10.12688/wellcomeopenres.26136.1)
Supplement: Peer review history

# Reviewer report: Reviewer response for version 1

**DOI:** 10.21956/wellcomeopenres.28783.r152947

**Published:** 2026-05-07

**Authors:** Lindsey C Perkin

**Affiliations:** 1 USDA, Agricultural Research Service, College Station, Texas, USA

The authors describe the genome sequence of the cabbage seedpod weevil, a pest of several agricultural crops. The authors use three weevils to collect data - DNA, Hi-C, and RNA. The methods are described well and consistent with current genome standards. I was able to access the data using the link provided. The Darwin Tree of Life group always does a thorough job in describing the protocol and linking to cites with very detailed protocols. 

 The results are a high quality genome and annotations.

Are sufficient details of methods and materials provided to allow replication by others?

Yes

Is the rationale for creating the dataset(s) clearly described?

Yes

Are the datasets clearly presented in a useable and accessible format?

Yes

Are the protocols appropriate and is the work technically sound?

Yes

Reviewer Expertise:

genomics, transcriptomics, comparative genomics, RNA-seq

I confirm that I have read this submission and believe that I have an appropriate level of expertise to confirm that it is of an acceptable scientific standard.

# Reviewer report: Reviewer response for version 1

**DOI:** 10.21956/wellcomeopenres.28783.r152949

**Published:** 2026-05-06

**Authors:** Pablo Duchen

**Affiliations:** 1 Johannes Gutenberg-Universitat Mainz, Mainz, Rhineland-Palatinate, Germany

This manuscript presents the genome of *Ceutorhynchus obstrictus. *I think the paper is well written and its content will be very welcome in the community. Even if the methods seem sound I think this paper is lacking some additional explanations to back up their conclusions. Actually, I don't think any conclusions are presented at all. I'm not expecting a separate section just for this, but at least a bit more detailed descriptions and justifications of the methods used and the results obtained. Please read below for details.

 Introduction:

 This reads well, but I'm missing a paragraph with the justification of the study, including what genomes are already available for the genus or the tribe, taxonomically how far are the closest full genomes and to which species they belong, has there been any other genomic studies on this species, and (as usual) the biological relevance of adding this genome. Please include the relevant literature as well.

 Methods:

 For the genome assembly part please add a few words for each program option and why was it chosen. For instance, you clearly stated that the -break option in YaHS handles potential misassemblies. This is clear, so you could use this as a template for other options. For example, what does the -primary option in Hifiasm do? What kind of "evaluation" does Gfastats and MRQURY.FK does?

 In the Assembly curation part please explain clearly how exactly "manual curation" works in your case. How did you "visually inspected and corrected" scaffolds? How did you decide what to break and what to join?

 Genome sequence report:

 In the legend of Figure 2 (and in the text) please explain a bit more in detail how can you conclude a genome size of 710Mb based on the shape of the plot.

 Could you please add an explanation or a conclusion of the k-mer completeness analysis? (if possible also add such explanation to the legend of Fig. 4).

 Citations of Figures 5 and 6 are very uninformative. They don't provide any explanation or conclusion from these figures.

 I think Table 5 could go as a supplementary Table. Instead, a table showing chromosome information, including names, and lenghts (from Fig. 3), as well as gene annotation information would be more useful.

Are sufficient details of methods and materials provided to allow replication by others?

Partly

Is the rationale for creating the dataset(s) clearly described?

Yes

Are the datasets clearly presented in a useable and accessible format?

Yes

Are the protocols appropriate and is the work technically sound?

Yes

Reviewer Expertise:

Genomics, Population Genomics, Systematics, statistical modelling.

I confirm that I have read this submission and believe that I have an appropriate level of expertise to confirm that it is of an acceptable scientific standard, however I have significant reservations, as outlined above.

# Reviewer report: Reviewer response for version 1

**DOI:** 10.21956/wellcomeopenres.28783.r152951

**Published:** 2026-04-22

**Authors:** Liang Lü

**Affiliations:** 1 Hebei Normal University, Shijiazhuang, Hebei, China

I have reviewed the manuscript titled "The genome sequence of the cabbage seedpod weevil, Ceutorhynchus obstrictus (Marsham, 1802) (Coleoptera: Curculionidae)".

 The manuscript provides a high-quality, chromosome-level reference genome for Ceutorhynchus obstrictus. Given the economic importance of this species as a pest of oilseed rape (Brassica napus), this genomic resource is timely and valuable for studies on pest management, insecticide resistance, and evolutionary biology within the Curculionidae family. The assembly statistics (N50, BUSCO scores) indicate a high level of completeness and continuity.

 Specific Comments on Methodology

 1. Specimen Selection and Identification

 The study used a single male specimen collected from Wytham Woods, Oxfordshire. While using a single individual for PacBio HiFi sequencing is standard practice to reduce heterozygosity complications, the authors should clarify if any voucher specimens (or the remains of the sequenced individual) are deposited in a museum to allow for future morphological verification, especially given the taxonomic complexity of the Ceutorhynchus genus. Notably, since the author did perform dissection and morphology-based identification, the manuscript would easily benefit from displaying images of aedeagus or more feature-bearing structures.

 2. Genome Assembly and Curation

 The authors should explicitly state if any major structural errors (e.g., large inversions or translocations) were identified and corrected during the manual curation phase, as this is often the most critical step for "chromosomal-level" claims.

 3. Sex Chromosome Identification

 The assembly identifies 14 chromosomal pseudomolecules, including X and Y. Identifying the Y chromosome in insects is often challenging due to its heterochromatic and repetitive nature. The authors should detail the evidence used to distinguish the Y chromosome from unplaced scaffolds or autosomal fragments, in particular when they used a single individual for Hi-C sequencing (male or female? Not mentioned). Was coverage analysis (half-coverage in males for X/Y) or homology to known Curculionidae sex-linked markers used?

 4. Annotation Quality

 Although the authors mentioned there were three specimens used for RNA sequencing and RNA library preparation, the methodology does not mention the use of species-specific RNA-seq data for evidence-based annotation. If RNA-seq was not used, the authors should acknowledge that the gene models are primarily based on ab initio predictions and homology, which may affect the accuracy of isoform detection.

 5. Mitochondrial Genome

 A 22.73 kb mitochondrial genome was assembled (either not annotated). This is significantly larger than the typical 15–18 kb insect mitogenome. The authors should clarify if this length is due to a large A+T-rich control region or if there are assembly artifacts (e.g., tandem repeats or circularization overlaps) that were not trimmed.

 Recommendation

 Accept with minor revisions. The methodology is technically sound and follows the consistent standards of the Darwin Tree of Life project. Addressing the points regarding heterozygosity, sex chromosome identification evidence, mitochondrial length, and especially annotation would improve the manuscript's utility for the broader entomological community.

Are sufficient details of methods and materials provided to allow replication by others?

Partly

Is the rationale for creating the dataset(s) clearly described?

Yes

Are the datasets clearly presented in a useable and accessible format?

Yes

Are the protocols appropriate and is the work technically sound?

Yes

Reviewer Expertise:

Phylogenomics and taxonomy of beetles

I confirm that I have read this submission and believe that I have an appropriate level of expertise to confirm that it is of an acceptable scientific standard.
